# Efficiency of a novel vertebral body augmentation system (Tektona™) in non-osteoporotic spinal fractures

**DOI:** 10.1186/s12891-022-05272-2

**Published:** 2022-04-13

**Authors:** Laura Marie-Hardy, Yann Mohsinaly, Raphaël Pietton, Marion Stencel-Allemand, Marc Khalifé, Raphaël Bonaccorsi, Nicolas Barut, Hugues Pascal-Moussellard

**Affiliations:** 1 Orthopaedic Surgery Department, Spine Unit; Pitié-Salpétrière Hospital, 47-83 bd de l’hôpital, 75013 Paris, France; 2 Orthopaedic Surgery Department, Spine Unit; Hopital Européen Georges Pompidou, 20 rue Leblanc, 75015 Paris, France

**Keywords:** Biomechanical, Cement, Compression fracture, Vertebral body fracture

## Abstract

**Background:**

The restauration of the local kyphosis is crucial to thoracolumbar fractures outcomes. Recently, the Tektona™ (Spine Art) system, constituted by a flexible lamella for corporeal reduction has emerged as a promising solution for osteoporotic fractures. However, no study has yet focused on its results on traumatic fractures.

**Methods:**

A retrospective longitudinal study on prospectively collected data was conducted on 53 patients that had a kyphoplasty by Tektona™, associated or not to percutaneous fixation. The data collected were clinical, surgical and scannographic (measurement of AVH, MVH and PVH (anterior/medium/posterior vertebral height), and RTA (regional traumatic angle) in°), preoperatively, post-operatively and at last follow-up.

**Results:**

Fractures were mainly located at the upper lumbar spine and were AOSpine A3 type for 74%. The mean RTA was 12° in pre-operative, 4° in post-operative (*p = 2e*^*− 9*^), and 8° at the last follow-up (*p = 0,01*). The mean correction of RTA for the fixation group was − 10 ± 6° versus − 7 ± 4° for the kyphobroplasty alone group *(p = 0,006).* The mean correction for fractures located at T10-T12 was − 9 ± 3°, − 9 ± 5° for L1, − 8 ± 3° for L2 and − 5 ± 3° for L3-L5 *(p = 0,045).*

**Conclusions:**

The Tektona® system appears to be efficient for acute thoraco-lumbar fractures, comparable to other available systems, allowing a real intracorporeal reduction work. Its relevance, especially in the long term needs further investigation. The association of a percutaneous fixation allow to obtain a better correction of the RTA but did not seem to prevent the loss of correction at follow-up.

**Supplementary Information:**

The online version contains supplementary material available at 10.1186/s12891-022-05272-2.

## Background

Traumatic vertebral fractures are a matter of public health, considering its incidence and the disability induced, notably in terms of return to work and back pain [1]. In AOSpine A fractures, the restoration of the sagittal alignment appears to be crucial [2, 3]. Kyphoplasty as a treatment of AOSpine A fractures has been at first described by Belkoff et al. and used initially in metastatic patients, before an exponential use in osteoporotic fractures, replacing cimentoplasty in some indications [4–6]. Kyphoplasty has also been used as an alternative to reduction and fixation in AOSpine A traumatic fractures, both on osteoporotic and non-osteoporotic patients [7–11]. The goals of a kyphoplasty for traumatic AOSpine A fractures are the restoration of the vertebral shape and the local kyphosis, with a mini-invasive treatment. Its advantage over internal fixation may therefore be the reduction of complications related to the instrumentation (rob breakage, infection, screw loosening…) and leaving motility to the discs adjacent to the fracture [10, 11]. In AOSpine A3 and A4 fractures, for which corporeal reduction is crucial, kyphoplasty may therefore be a mini-invasive solution to avoid anterior approaches, their scarring ransoms, and potential complications. Additionally, AOSpine A fractures have been described as strictly bony fractures, and the proper reduction of the vertebral body reduction seems to reduce the risk of secondary disc degeneration [12, 13]. However, most of the available devices only allow one trial at corporeal reduction and do not allow to target the area to be reduced depending of the fracture characteristics.

A decade ago, a new vertebroplasty solution has been launched. The Tektona™, (Spine Art) system is a percutaneous vertebral body reduction system. It is constituted by a flexible lamella, that can be orientated in several plans and used several times in the same procedure into the vertebral body, to reduce the fracture and the traumatic vertebral kyphosis. However, this new device has, at our knowledge, only been reported in two studies. The first one, by Krüger et al. was a cadaveric study, comparing the Tektona® to a standard balloon kyphoplasty and concluded to promising results [14]. The second one, by Marcia et al. described a cohort of 30 patients, with encouraging results in terms of clinical scores and vertebral body height (VBH) restoration but only on an osteoporotic population [15]. Is the Tektona reliable in terms of sagittal correction on acute vertebral fractures? Are the results of this new technic with a flexible lamella t be comparable to other existing devices?

The main goal of this study was to measure the efficiency, in term of vertebral body reduction of the Tektona®, on a non-osteoporotic spinal fracture cohort.

## Methods

### Patients

**Fig. 1:**
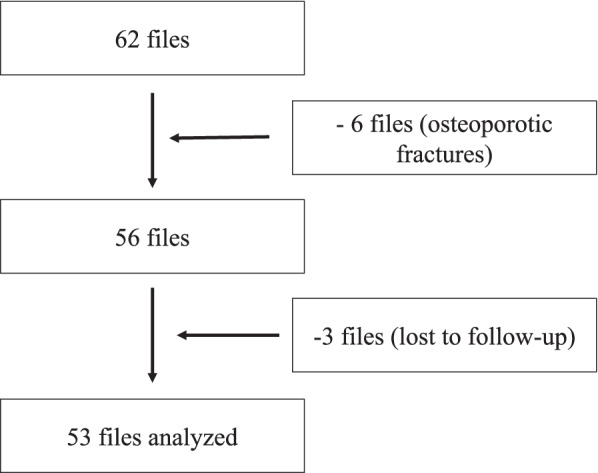
Flow-chart of the study

The files of all patients treated in a single spinal surgery center between January 2015 and April 2021 treated by Tektona vertebral body augmentation system have been systematically reviewed. This was a retrospective longitudinal study on prospectively collected data. This study was subject to a privacy impact assessment (PIA) by the data protection office BPD2018DIA002 that waived the need for ethical committee approval and registered as an observational study in the APHP registry with the number 20220107154410. All analyses were performed in accordance with the principles of the Declaration of Helsinki. The files of 53 patients have been included. The flow-chart is available as Fig. 1.

The inclusion criteria were:

- AOSpine A vertebral body fracture, between T7 and L5
- Patients over 18 years and under 65 years
- Pre-operative CT-scan at the fracture level and early (< 3 months) and at follow-up postoperative CT-scan (before the instrumentation removal).

The exclusion criterias were:

- Osteoporotic fractures defined as bone mineral density in L1 < 150HU (Hounsfield Unit) [16–18].
- Lack of follow-up.

### Surgical technic

#### Surgeries were realized by specialized spinal surgeons

**Fig. 2:**
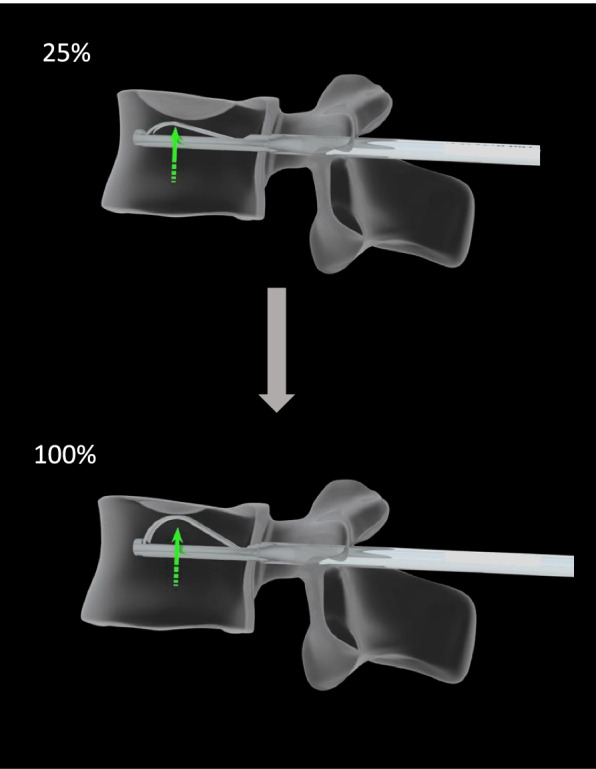
Scheme illustration the flexible lamella of the Tektona system progressive bend allowing to control the strength of reduction needed

**Fig. 3:**
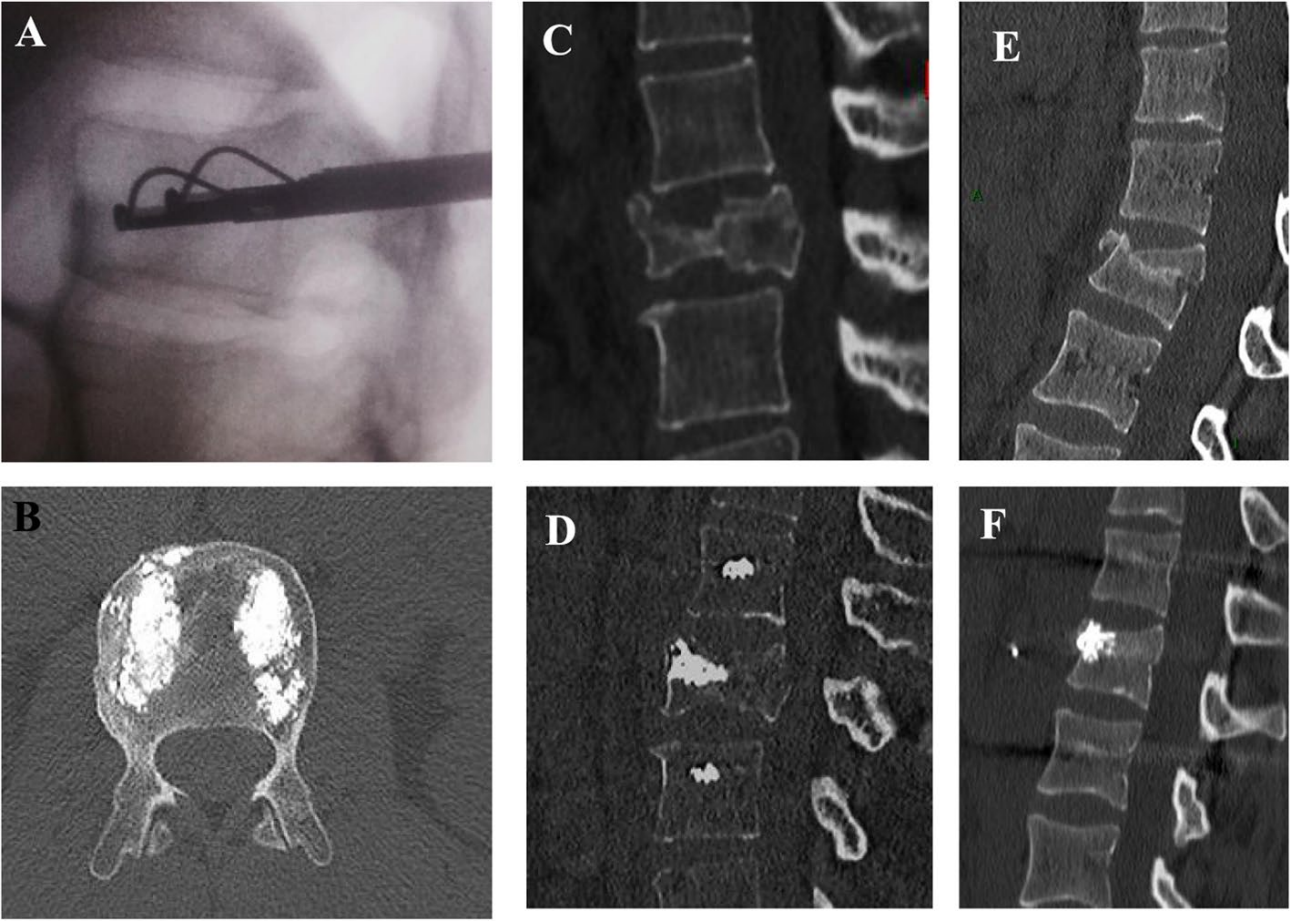
**A**: Per-operative C-arm imaging of the reduction of the superior endplate by the flexible lamella. **B**: Axial CT-scan view of vertebral body cementing. **C** and **D**: Same patient, pre- and postoperative sagittal CT-scan imaging after a vertebroplasty by Tektona™. E and F: Same patient, pre- and postoperative sagittal CT-scan imaging after a vertebroplasty by Tektona™

All surgical indications were approved by a specialized surgical meeting. Additional percutaneous fixation was plebiscite for fractures presenting risk factors of failure for the kyphoplasty alone, as an important kyphotic deformity, localized at the thoraco-lumbar junction and classified AOSpine A4 or A3. All patients were operated in prone position, under fluoroscopic guidance (C-arm). Jamshidi were introduced in both pedicle of the fractured vertebra, then replaced by K-wires, to allow the introduction of the working cannula. The reduction instrument was then inserted into the vertebral body. The vertebral reduction was obtained with the progressive flexion of the flexible lamellas. The placement of the lamella under the fracture was controlled by fluoroscopy. The reduction movement was repeated until a satisfying image was obtained, corresponding firstly to a reduction of the superior vertebral endplate anteriorly, medially, and posteriorly; secondly to a minimal reduction of the medial depression of 50% of the initial defect; and thirdly to a stable reduction with no major loss of correction at the device removal. The Fig. 2 illustrate the progressive reduction that can be achieved by the increase of the lamella bend. The cement (PMMA, Mendec Spine HV System, Tecres, Italie) was then inserted in the space thus created by the reduction. An example of per-operative use is displayed as Fig. 3. When additional fixation was needed, bilateral percutaneous pedicle fixation from one level below the fractured level to one level above (1 + 1), was realized after the kyphoplasty, during the same operating time.

### Data

The data collected were demographic, clinical, surgical, and radiologic. The demographic data collected for all patients were age, sex and the delay between the trauma and the surgery. The clinical data related to the fractures collected were: level, type according to AOSpine classification [19]. The surgical data collected were the delay between the trauma and the surgery, the level of the vertebroplasty, the association to a percutaneous fixation and the surgical complications.

The scannographic study of the patients comprised, pre-operatively, at early and last- follow-up, according to the department protocol for all vertebral fractures, the measurement of AVH (anterior vertebral height) in millimeters (mm), MVH (anterior vertebral height) in mm, PVH (posterior vertebral height), and TRA (traumatic regional angle) in°. The TRA was defined as regional kyphosis (RK) – physiological regional angle (PRA) defined by Guigui et al. [20, 21]. The regional kyphosis was according to the Cobb angle method. The HU measurements of bony density were realized on the pre-operative CT-scans using Carestream. The region of interest (ROI) was chosen over the trabecular vertebral body and the mean HU value of the ROI, avoiding lesion that may distort the measurement as focal lesions or venous plexus, was calculated on the L1 vertebrae, or L2 when L1 was fractured [16–18]. *All measurements were realized on Carestream® software by two senior surgeons.*

### Statistics

Values of AVH, MVH, PVH and TRA were compared pre-operatively and at early and last follow-up. A sub-group analysis between patients with associated fixation and without was also realized. The statistical analysis was realized on Excel Stat. Student t-test were used to compare series, *p* < 0,05 was considered as statistically significant.

## Results

### Cohort description

Among the 53 patents analyzed, 36 patients (68%) were male and 17 (32%) were female. The mean age at the time of the surgery was 41 ± 14 years; [18;64]. The mean delay between the trauma and the surgery was 2,3 ± 1,7 days; [0;10]. All patients had an early post-operative CT scan, whereas only 29 had a late CT-scan, at a mean follow-up of 17 months. The mean pre-operative bony density was 223 ± 42; median value: 205; [min-max: 167–384] HU.

**Fig. 4:**
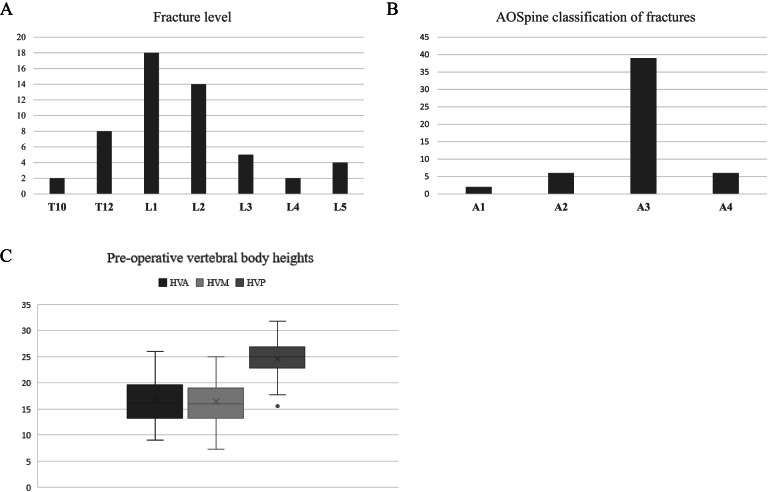
**A**: Histogram showing the repartition of the fracture’s levels. **B**: Histogram showing the repartition of the fracture’s types according to AOSpine’s classification. C: Box plots showing the vertebral body height (AVH: anterior vertebral height, MVH medium vertebral height and PVH: posterior vertebral height), in millimeters, in pre-operative

Fractures were predominantly located at the upper lumbar spine: L1 for 18 patients (34%) and L2 for 14 patients (26%), Fig. 4A. Most fractures were A3 according to AOSpine classification (39 patients, 74%) (Fig. 4B). Twenty patients (38%) had a percutaneous fixation including one level below the fractured level and one level above (1 + 1 fixation) associated to the kyphoplasty.

Vertebral loss of height was mainly anterior and median (Fig. 4C). The relative anterior body height compared to the posterior was equal to 70 ± 4,3% [46, 104] and 66 ± 3,8% [41;93] for the relative medium body height.

Complications occurred in 5 patients (10%), consisting in 3 anterior cement leaks and 2 posterior cement leaks. None of these patients were symptomatic and no surgical re-intervention had to be performed. No surgical revision due to surgical site infection has to be performed. No per-operative complication due to the device (eg. lamella breakage) was observed.

### Radiological results

**Fig. 5:**
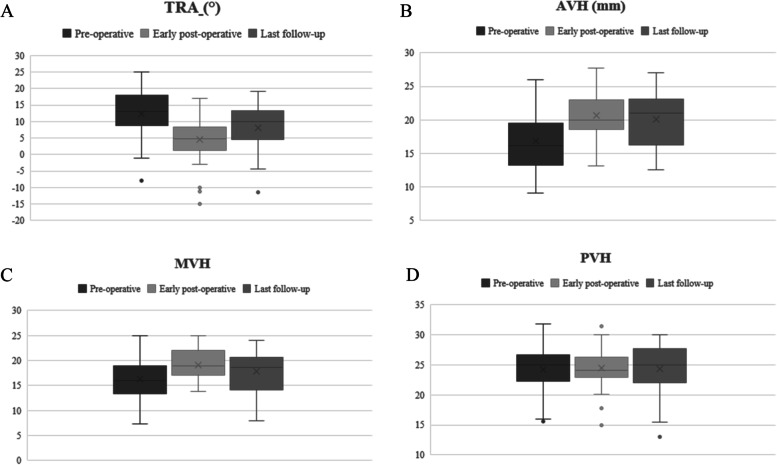
**A**: Box plots showing the traumatic vertebral angle, in °, at the 3 times of analysis (preoperative, early post-operative and at follow-up). **B**: Box plots showing the AVH, in mm, at the 3 times of analysis (pre-operative, early post-operative and at follow-up). **C**: Box plots showing the MVH, in mm, at the 3 times of analysis (pre-operative, early post-operative and at follow-up). **D**: Box plots showing the PVH, in mm, at the 3 times of analysis (pre-operative, early post-operative and at follow-up)

The mean RTA was 12° in pre-operative, 4° in post-operative and 8° at the last follow-up. The difference between pre-operative and early post-operative results were statistically significant (*p = 2e*^*− 9*^), as well as the difference between early and late RTA (*p = 0,01*), (Fig. 5A).

The mean evolution between pre-operative and early post-operative AVH was + 27; ±30%; [− 19; 131], (*p = 2e*^*− 6*^) and between early and late post-operative was − 6; ±13%; [− 32; + 29], (*p = 0,20*), (Fig. 5B).

The mean evolution between pre-operative and early post-operative MVH was + 15; ±13%; [− 23; 57], (*p = 7e*^*− 5*^) and between early and late post-operative was − 12; ±18%; [− 75; + 13], (*p = 0,16*), (Fig. 5C).

The mean PVH was not different between the 3 times of analysis: 24,3 ± 3,3° in pre-operative, 25 ± 2,9° in early post-operative and 24 ± 4,2° in late post-operative (*p = 0,7*), (Fig. 5D).

The mean correction of RTA for the sub-group of patients that had a percutaneous associated fixation (fixation group) was − 10 ± 6° versus − 7 ± 4° for the kyphoplasty alone group. This difference was statistically significant *(p = 0,006).* At last follow-up, the loss of correction for the sub-group of patients that had a percutaneous associated fixation (fixation group) was + 3,7° ± 7° versus + 3,8 ± 8° for the vertebroplasty alone group; this difference was not statistically significant *(p = 0,97).*

The mean correction for fractures located at T10-T12 was − 9 ± 3°, − 9 ± 5° for L1, − 8 ± 3° for L2 and − 5 ± 3° for L3-L5. There were a statistically significant difference of correction for fracture below L2 *(p = 0,045).*

## Discussion

These results show a clear reduction of the RTA obtained by Tektona® vertebroplasty, with or without associated percutaneous fixation, for AOSpine A fracture between T10 and L2. The loss of correction at follow-up was measured at 4°, after obtaining 8° of RTA reduction by the procedure. The anterior vertebral body height seemed better reduced by the procedure than the medium superior vertebral endplate depression (+ 27; ±30% versus + 15; ±13%). The association of a percutaneous fixation allowed to obtain a better correction of the RTA but did not seem to prevent the loss of correction at follow-up.

The 8° RTA restoration post-operatively is comparable to other series of traumatic fractures treated by kyphoplasty in the literature (− 4° for Garnon et al.; − 11° for Maestrettri et al.; − 3° for Hartmann et al.; − 6° for Teyssédou et al.) [9–11, 22]. The advantage of the Tektona® reduction system seems to lie in the design itself. Indeed, to reduce the endplate depression, one need to apply the principles of general traumatology, trying all needed maneuver to obtain the maximum correction of the endplate depression. However, to date, none of the kyphoplasty devices allows to have an oriented, repeatable, reduction maneuver. The Tektona® system is the first device allowing to do so, even in the rare inferior end plate traumatic fracture (by a 180° rotation of the device). However, comparisons to other existing kyphoplasty solutions have still to be performed in various parameters. For example, in a meta-analysis by Jing et al. single balloon have been proven as effective as double balloon bipedicular kyphoplasty, with an obvious cost advantage [23]. Will the results be similar with the Tektona system?

The system also needs to be compared to others existing device regarding complications. The rate of cement leakage (asymptomatic) was 10% in this study and 16% in the work of Marcia et al., that may seems lower that reported for other devices, as 25,5% for the Vertebral Body Stenting® (VBS) (Synthes, Soletta, Switzerland), and up to 39% for the SpineJack (Vexim, SA, Balma, France) system [15, 24, 25].

The loss of correction (4°) at a mean follow-up of 17 months is also comparable to the results presented in these studies. The Tektona system seems to permit a satisfying correction of the AVH (+ 27; ±30%), while the correction of the medium body height seems perfectible (+ 15; ±13%). The correction of the middle vertebral body height remains indeed one of the main challenges, especially in burst fractures. Another highlight of the study is that if these results are promising for fractures between T10 and L2, the results are more mitigated for fracture below L2. That may be explained by the higher among of axial load in the lower lumbar spine. However, surgeon must be aware of the more mitigated results below L2 and carefully choose the indication of kyphoplasty in this region.

There are some limitations to this study. The retrospective design of the study indeed comprises inherent bias, *as well as the lack of a control group*. The quantity of cement injected would have been an interesting data to correlate to the loss of correction. The lack of clinical data is regrettable, but clinical results seems to correlate with sagittal angulation in spinal fractures, at early as well as in long-term perspective [26, 27].

## Conclusions

In conclusion, the Tektona® system seems to be an interesting device for acute fractures between T10 and L2, with a correction that seems comparable to other available systems, but promising possibilities of technical use development.

## Supplementary Information

**Additional file 1.**

## Data Availability

The data are available as supplementary material.

## Abbreviations

AVH: Anterior vertebral height
CT: Computed tomography
MVH: Medium vertebral height
PVH: Posterior vertebral height
RTA: Regional traumatic angle
VBH: Vertebral body height
